# Intratumoral Translocation Positive Heterogeneity in Pediatric Alveolar Rhabdomyosarcoma Tumors Correlates to Patient Survival Prognosis

**DOI:** 10.3389/fcell.2020.564136

**Published:** 2020-09-18

**Authors:** Katrina Gleditsch, Jorge Peñas, Danielle Mercer, Ayesha Umrigar, James Briscoe, Matthew Stark, Fern Tsien, Andrew D. Hollenbach

**Affiliations:** ^1^Department of Genetics, Louisiana State University Health Sciences Center, New Orleans, LA, United States; ^2^Division of Pediatric Hematology/Oncology, Children’s Hospital of New Orleans, New Orleans, LA, United States; ^3^Department of Pathology, Children’s Hospital of New Orleans, New Orleans, LA, United States

**Keywords:** Alveolar rhabdomyosarcoma, tumor heterogeneity, prognostic outcome, Pax3-FOXO1, molecular genetics

## Abstract

Alveolar rhabdomyosarcoma (ARMS) is characterized by one of three translocation states: *t*(2;13) (q35;q14) producing PAX3-FOXO1, *t*(1;13) (p36;q14) producing PAX7-FOXO1, or translocation-negative. Tumors with *t*(2;13) are associated with greater disease severity and mortality than *t*(1;13) positive or translocation negative patients. Consistent with this fact, previous work concluded that a molecular analysis of RMS translocation status is essential for the accurate determination of prognosis and diagnosis. However, despite this knowledge, most diagnoses rely on histology and in some cases utilize fluorescence *in situ* hybridization (FISH) probes unable to differentiate between translocation products. Along these same lines, diagnostic RT-PCR analysis, which can differentiate translocation status, is unable to determine intratumoral translocation heterogeneity, making it difficult to determine if heterogeneity exists and whether correlations exist between this heterogeneity and patient outcomes. Using newly developed FISH probes, we demonstrate that intratumoral heterogeneity exists in ARMS tumors with respect to the presence or absence of the translocation product. We found between 3 and 98% of cells within individual tumor samples contained a translocation event with a significant inverse correlation (*R*^2^ = 0.66, *p* = 0.001) between the extent of intratumoral translocation heterogeneity and failure-free survival of patients. Taken together, these results provide additional support for the inclusion of the molecular analysis of these tumors and expand on this idea to support determining the extent of intratumoral translocation heterogeneity in the diagnosis of ARMS to improve diagnostic and prognostic indicators for patients with these tumors.

## Introduction

Rhabdomyosarcoma (RMS) is the one of the most common pediatric soft tissue sarcomas in the United States. Initial clinicopathologic phenotypes are determined histologically and are typically characterized by two main subtypes–alveolar (ARMS) and embryonal (ERMS) (Parham and Barr, 2013). Of these two subtypes, ARMS has a more aggressive clinical course and a poorer prognosis with a 4-year failure-free survival rate for patients with localized and metastatic ARMS being 65 and 15%, respectively (Barr, 2009). ARMS is most commonly characterized by the presence of a *t*(2;13) (q35;q14) (~60%) or a *t*(1;13) (p36;q14) (~20%) chromosomal alteration, generating the PAX3-FOXO1 (Barr et al., 1993; Shapiro et al., 1993) or PAX7-FOXO1 (Davis et al., 1994) oncogenic fusion proteins, respectively. Clinically, *t*(2;13) containing ARMS tumors have been shown to be more aggressive than *t*(1;13) containing tumors (Anderson et al., 2001), requiring a more aggressive treatment protocol.

In addition to histological subtype and translocation status, additional factors have been reported to affect prognosis, including age at diagnosis, stage of tumor progression, clinical grade of the tumor (based on the extent of residual disease after surgery), location of the primary tumor, and the presence of metastasis (Lawrence et al., 1987; Crist et al., 1995, 2001; Raney et al., 2001). Despite this information, standard practice in the diagnosis of ARMS in the United States includes the determination of histological phenotype, immunohistochemistry to determine expression levels of muscle specific genes, and occasionally fluorescence *in situ* hybridization (FISH) to determine translocation status (Morotti et al., 2006). In the latter case the most commonly used FISH probe is the FOXO1 break apart probe (Balogh et al., 2016), which determines whether FOXO1, the common gene in both *t*(1;13) and *t*(2;13), is intact or has been “broken apart” as a result of a translocation event. While adequate to determine the presence of a FOXO1-containing genetic alteration, the use of this probe requires subsequent RT-PCR analysis to identify the exact translocation event, with RT-PCR being unable to provide more detail about the potential intratumoral heterogeneity of the translocation.

More recent literature evidence utilized FISH probes capable of simultaneously identifying both translocation events to demonstrate that neither the histology of RMS tumors nor the location of the primary tumor site had any influence on the response rate to chemotherapy (Burke et al., 2007). Information based on a meta-analysis of metagene signature expression determined that a majority of the prognostic value for RMS was dominated by fusion gene status (Missiaglia et al., 2012). Taken together, these studies strongly support placing a higher emphasis on the molecular (i.e., fusion gene) status rather than histopathological data in the risk stratification of these patients (Selfe et al., 2017).

In addition to fusion gene status, previous studies determined that significant clonal genetic heterogeneity with respect to gross chromosomal alterations is present within RMS tumor samples and that clinical outcome varied dependent on the extent of clonal genetic heterogeneity with respect to gross structural changes (Walther et al., 2016). These data showed that fusion positive ARMS contained more gross structural heterogeneity than other subtypes (Walther et al., 2016), which may be due, in part, to later evidence reporting that the presence of PAX3-FOXO1 is sufficient to promote aneuploidy and genomic instability (Loupe et al., 2016). However, despite the knowledge that translocation status and clonal genetic heterogeneity are important for determining patient prognosis, it has not yet been quantitatively determined if intratumoral heterogeneity exists in ARMS with respect to the presence or absence of the translocation and whether this extent of heterogeneity correlates to clinical outcomes.

In the present work we describe the use of FISH probes that allow the simultaneous determination of the presence or absence of *t*(1;13) and/or *t*(2;13) translocations in RMS. We use these probes to analyze a series of RMS tumors obtained from patients at the Children’s Hospital of New Orleans, Louisiana (CHNOLA). Interestingly, we found that intratumoral heterogeneity with respect to the presence or absence of translocation events within individual cells varied between ARMS tumor samples. Finally, a retrospective chart review determined that a significant inverse correlation exists between the number of translocation positive cells within a tumor and patient outcomes. The findings presented in this work provide additional evidence supporting the inclusion of molecular analyses that include the determination of not only the presence but also the extent of fusion gene status to improve diagnostic and prognostic indicators for patients with these tumors.

## Materials and Methods

The project was reviewed by both the Louisiana State University Health Sciences Center and the Children’s Hospital of New Orleans Institutional Review Boards and approved under IRB #8504. All patient information was protected according to HIPAA standards. Patient tumor samples were selected based on histology reports identifying tumors as rhabdomyosarcoma, as determined by the Pathology and Histology Departments at Children’s Hospital of New Orleans. Tumor samples were preserved in 10% formaldehyde neutral buffered solution and paraffin. Tissue blocks were cut using a microtome at 4 μm onto fresh slides and embedded with paraffin media. Slides containing the tissue samples were placed in a 60°C oven for 45 min. After baking, slides were placed in 100% xylene washes 3× for 5 min each, followed by hydration by placing the slides in 100% ethanol 3× for 2 min each, 1× in 95% ethanol for 1 min, and then a final wash in dH_2_O for 1 min, all at room temperature. Slides were then placed in a pressure cooker containing 10 mM citrate (pH 6.0) and incubated for 10 min at 120°C.

After removal from the pressure cooker, slides were washed two times for 3 min each with 1× phosphate buffered saline supplemented with Tween 20 (PBST), using a clean Coplin jar for each wash. After the slides dried, 500 μL of a Proteinase K solution (Abcam ab64220-Proprietary Concentration), preheated to 37°C, was placed onto the tissue area of the slide. Coverslips were placed over the slides to achieve uniform coverage of Proteinase K after which the slides were incubated at 37°C for 10 min. After incubation the slides were washed in PBST for 2 min and dehydrated for 1 min each using 70, 85, and 100% ethanol, consecutively.

Fluorescence *in situ* hybridization (FISH) was performed using a series of overlapping probes specific for PAX3, PAX7, or FOXO1 genomic loci, which were designed in collaboration with and purchased from Cytocell, Ltd. (Tarrytown, NY). The probe mixtures were comprised of a series of overlapping DNA segments specific for each genomic locus as follows: PAX3 (Chr2q36.1; 222,622,739–223,352,361), PAX7 (Chr1p36.13; 18,624,373–19,590,790), and FOXO1 (Chr13q14.11;40,409,578–42,030,365). Fluorochromes were chosen for each probe with specific excitations (ex) and emission (em) wavelengths: PAX3 (Orange, ex/em = 551/572), PAX7 (Aqua, ex/em–418/467), and FOXO1 (Green, ex/em = 495/521). DAPI (Abbott Molecular, Abbott Park, IL; ex/em = 350/460) was used as a counterstain to label cell nuclei.

The FISH probes were provided in a stock concentration of 2.5 ng/μL in buffer of formamide, dextran sulfate, sodium dodecyl sulfate (SDS), and 2× sodium citrate/sodium chloride (pH 7.0), formulated and optimized by Cytocell for the GC content of the probes. Four microliters of each probe stock were combined for a total of 12 μL (0.83 ng/μL final concentration for each individual probe). The probe mixture and the tissue samples were pre-warmed at 37°C for 5 min after which the probe mixture was pipetted onto the tissue slides. A coverslip was applied, sealed with rubber glue, and the sample and probe were denatured by placing the samples onto a heating block at 75°C for 5 min. Once denatured, slides were placed in a humidified hybridization chamber for 72 h at 37°C. After incubation the non-hybridized probes were removed by washing with a 2× SSC (pH 7.0), 0.3% NP-40 buffer at 74°C for 2 min followed by a second wash of 2× SSC (pH 7.0) at room temperature. Slides were dried after which 12 μL of DAPI I (1,000 ng/μL) was pipetted onto slides, covered with a coverslip, and placed at -4°C until analyzed.

Tumor samples were analyzed at 10× and 100× magnification using an Olympus Bx60 microscope with fluorescent capabilities and a cooled CCD camera connected to the Leica CytoVision Automated Cytogenetics Platform imaging software. Fluorescence filters from Leica Biosystems were consistent with the excitation and emission wavelengths specific for each probe. A minimum of 100 images were captured and examined for each tumor sample and analysis of translocation status was performed with the observer being blinded to the clinical data.

## Results

**FIGURE 1:**
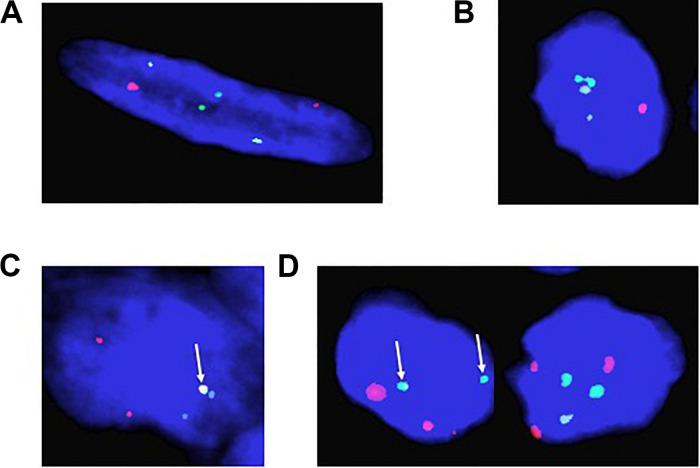
Representative fluorescence *in situ* hybridization results for **(A)** normal aged-matched muscle control; **(B)** embryonal rhabdomyosarcoma (ERMS); **(C)** *t*(2;13) positive Alveolar rhabdomyosarcoma (ARMS); and **(D)** *t*(1;13) positive Alveolar rhabdomyosarcoma (ARMS). Red = PAX3; Green = FOXO1; and Aqua = PAX7. The arrows indicate the presence of a translocation event.

Fifty-five rhabdomyosarcoma patient tumor samples were received from Children’s Hospital in New Orleans, all samples being obtained from a tumor bank derived from biopsies performed over the previous 30 years. We used aged-matched normal muscle tissue derived from autopsies as a control. We performed FISH analysis on all samples using our probes for PAX3, PAX7, and FOXO1 analyzing 100 individual cells for each sample. Of the original 55 samples, tumors from 37 patients were of sufficient quality to provide usable data. We observed two distinct signals for PAX3 (red), PAX7 (aqua), and FOXO1 (green) in the normal muscle control samples, which is consistent with the presence of two alleles for each gene (Figure 1A). We also observed distinct signals for each of the genes in 25 of the 37 samples (Figure 1B), indicating that these samples were translocation negative for the chromosomal regions in question (i.e., fusion negative). Of these samples, 14 were originally histologically categorized as ERMS, 7 as ARMS, with the remaining 4 being of unknown or undetermined histological categorization (Table 1).

**TABLE 1:** Clinical and cytogenetic status of RMS patient tumor samples.

Initial biopsy^*a*^	Translocation^*b*^	Staging^*c*^	Metastasis^*c*^	Age at diagnosis^*c*^	Percent aneuploid^*b*^ (%)	Survival^*c*^
ARMS	Negative	–^*e*^	–	10Y 0M	79	A (14.00Y)^*d*^
ARMS	Negative	–	–	4Y 1M	11	A (16.00Y)
ARMS	Negative	3	No	2Y 2M	31	A (18.54Y)
ARMS	Negative	–	–	14Y 2M	85	D (2.65Y)
ARMS	Negative	–	–	1Y 9M	10	D (3.08Y)
ARMS	Negative	4, CG4	Yes	1Y 7M	16	D (4.33Y)
ARMS	Negative	–	–	3Y 9M	6	D (6.25Y)
ERMS	Negative	4, CG4	Yes	1Y 7M	72	A (5.83Y)
ERMS	Negative	1, CG2B	Yes	16Y 2M	47	A (9.00Y)
ERMS	Negative	–	No	2Y 6M	0	A (10.25Y)
ERMS	Negative	–	–	7Y 6M	66	A (11.00Y)
ERMS	Negative	4, CG4	Yes	3Y 2M	67	A (11.01Y)
ERMS	Negative	–	–	2Y 11M	53	A (12.08Y)
ERMS	Negative	1, CG1	No	2Y 10M	43	A (12.75Y)
ERMS	Negative	3, CG3	No	2Y 2M	12	A (16.98Y)
ERMS	Negative	–	–	0Y 4M	6	A (22.33Y)
ERMS	Negative	4, CG4	Yes	3Y 2M	94	A (3.33Y)
ERMS	Negative	–	–	4Y 6M	33	D (2.17Y)
ERMS	Negative	–	–	2Y 6M	37	D (3.66Y)
ERMS	Negative	4, CG4	Yes	14Y 1M	3	D (6.83Y)
ERMS	Negative	–	Yes	3Y 3M	30	D (17.00Y)
Unknown	Negative	1, CG2A	No	13Y 5M	7	A (3.25Y)
Unknown	Negative	–	–		22	–
Unknown	Negative	–	–		7	–
Unknown	Negative	–	–		78	–
ERMS^*f*^	Positive *t*(1;13)	–	–	5Y 7M	54	A (14.91Y)
ARMS	Positive *t*(2;13)	3, CG3	No	16Y 5M	12	A (7.25Y)
ARMS	Positive *t*(2;13)	3, CG3	No	9Y 9M	74	A (11.03Y)
ERMS^*f*^	Positive *t*(2;13)	4, CG4	Yes	2Y 2M	86	A (4.89Y)
ARMS	Positive *t*(2;13)	–	–	1Y 0M	62	D (2.93Y)
ARMS	Positive *t*(2;13)	4, CG4	Yes	1Y 6M	65	D (1.49Y)
ARMS	Positive *t*(2;13)	4, CG4	Yes	8Y 10M	63	D (0.88Y)
ARMS	Positive *t*(2;13)	–	Yes	14Y 7M	62	D (1.65Y)
ARMS	Positive *t*(2;13)	4, CG4	Yes	17Y 5M	9	D (1.37Y)
ARMS	Positive *t*(2;13)	4, CG4	Yes	18Y 9M	82	D (1.48Y)
ARMS	Positive *t*(2;13)	–	–	18Y 9M	21	D (0.75Y)
ARMS	Positive *t*(2;13)	–	–	2Y 9M	25	U (4.57Y)

**^*a*^Tumor samples were received from Children’s Hospital in New Orleans, who performed the initial biopsy using standard histological methods. ^*b*^Translocation status and percent cells aneuploid for chromosomes 1, 2, and 13 were determined using our novel FISH probes, as described in the section “Materials and Methods.” ^*c*^Staging, metastasis, relapse, and failure free survival were determined from a retrospective chart review with patients being classified as Deceased (D) or Alive (A). ^*d*^Time in years (Y) between initial diagnosis to death or to last scheduled appointment are indicated in the parentheses. ^*e*^Dashes indicate either the patient was lost to follow up or that the original records were lost in the aftermath of Hurricane Katrina. ^*f*^Indicates tumor samples originally histologically characterized as ERMS but were determined to be translocation positive by FISH analysis.**

In the 12 remaining tumor samples we observed red/green fusion for PAX3 (red) and FOXO1 (green) (Figure 1C), consistent with a *t*(2;13). We also found one tumor sample containing aqua/green fusion for PAX7 (aqua) and FOXO1 (green) (Figure 1D), consistent with a *t*(1;13). Interestingly, 2 of these 12 translocation positive tumor samples were originally histologically categorized as ERMS with the remaining ten originally being categorized as ARMS (Table 1). The translocation positive samples derived from patients ranging in ages 1.0–18.8 years, with an age distribution consistent with previously reported age demographics for patients with ARMS (Ognjanovic et al., 2009). Of the 12 patient samples, 9 were female, 6 were African American, 3 were White/Caucasian, and 3 patient samples were of unreported racial/ethnic status or whose clinical records were lost in the aftermath of Hurricane Katrina. The primary tumor sites include the abdomen, lung, tibia, perineum, chest, head/neck, forearm, and testes.

Surprisingly, in addition to determining the translocation positivity within tumor samples, we observed that not all cells within a given fusion-positive tumor sample contained a translocation event (see Figure 1D). This intratumoral heterogeneity with respect to the presence or absence of a translocation event ranged from only 3% of cells in the tumor containing the *t*(1;13) to between 12 and 98% of cells containing the *t*(2;13) (Table 2). The non-translocation positive cells are histologically identical to the cells containing the translocation, indicating that the observed heterogeneity does not result from the presence of non-cancerous cells within the tumor. Further, our FISH analysis also identified the presence of multiple copies of the PAX3, PAX7, and FOXO1 loci (Figures 1B,D), which is technically considered aneusomy but referred to as aneuploidy within clinical diagnostics. Therefore, we will use the term aneuploidy in the remainder of this manuscript to maintain consistency with the clinical notation. Similar to our results for the presence or absence of the translocation, we also noted intratumoral heterogeneity with respect to the presence or absence of aneuploidy within individual cells. The percentage of cells containing a non-diploid number of chromosomes 1, 2, and/or 13 ranged from between 0 and 94% between different tumor samples regardless of whether the tumors were histologically characterized as ERMS, fusion-negative ARMS, or fusion-positive ARMS (Table 1).

**TABLE 2:** Clinical status and percent intratumoral translocation heterogeneity in translocation positive ARMS patients.

Patient ID	Original Biopsy^*a*^	*t*(2;13)^*b*^ (%)	*t*(1;13)^*b*^ (%)	Survival^*c*^
K	ERMS^*e*^	–	3	A (14.91Y)^*d*^
C	ARMS	12	–	A (7.25Y)
F	ARMS	16	–	A (11.03Y)
J	ARMS	33	–	D (2.93Y)
D	ARMS	68	–	D (1.49Y)
E	ARMS	71	–	D (0.88Y)
I	ARMS	75	–	D (1.65Y)
B	ARMS	76	–	D (1.37Y)
L	ARMS	79	–	D (0.75Y)
G	ARMS	79	–	U (4.57Y)
H	ARMS	94	–	D (1.48Y)
A	ERMS^*e*^	98	–	A (4.89Y)

**^*a*^Tumor samples were received from Children’s Hospital in New Orleans, who performed the initial biopsy using standard histological methods. ^*b*^Translocation status was determined using our novel FISH probes, as described in the section “Materials and Methods.” ^*c*^Failure free survival was determined from a retrospective chart review with patients being classified as Deceased (D) or Alive (A). ^*d*^The time in years between initial diagnosis to death or to last scheduled appointment is indicated in the parentheses. ^*e*^Indicates tumor samples originally histologically characterized as ERMS but were determined to be translocation positive by FISH analysis.**

We performed a retrospective chart review on the 37 samples to examine patient outcomes, including stage of tumor, clinical grade of tumor (which is based on the extent of the tumor and how completely it is removed during initial surgery), presence of metastasis at initial diagnosis, patient relapse, and failure-free survival in years. We included all data from available clinical records, with some records from before 2005 being lost in the aftermath of Hurricane Katrina. Consistent with the more aggressive nature of the tumor we saw a majority of patients initially diagnosed with fusion positive ARMS presented with higher grade of tumor (5/7 patients with available clinical data being stage 4, clinical grade 4) with metastasis to distant sites (6/8 patients with available clinical data). 7/12 patients with fusion positive ARMS succumbed to the disease, giving a higher mortality rate for these patients relative to fusion negative ARMS or ERMS (Tables 1, 2). In contrast, the patients with fusion negative tumors (either ERMS or ARMS) initially presented with a variety of tumor stages and grades (from stage 1, clinical grade1 to stage 4, clinical grade 4) and a lower incidence of metastases. Regardless of stage and clinical grade or presence of metastasis at initial presentation, fusion negative RMS patients had a lower mortality rate (8/21; 38%) relative to fusion positive ARMS (8/12; 67%) (Table 1).

**FIGURE 2:**
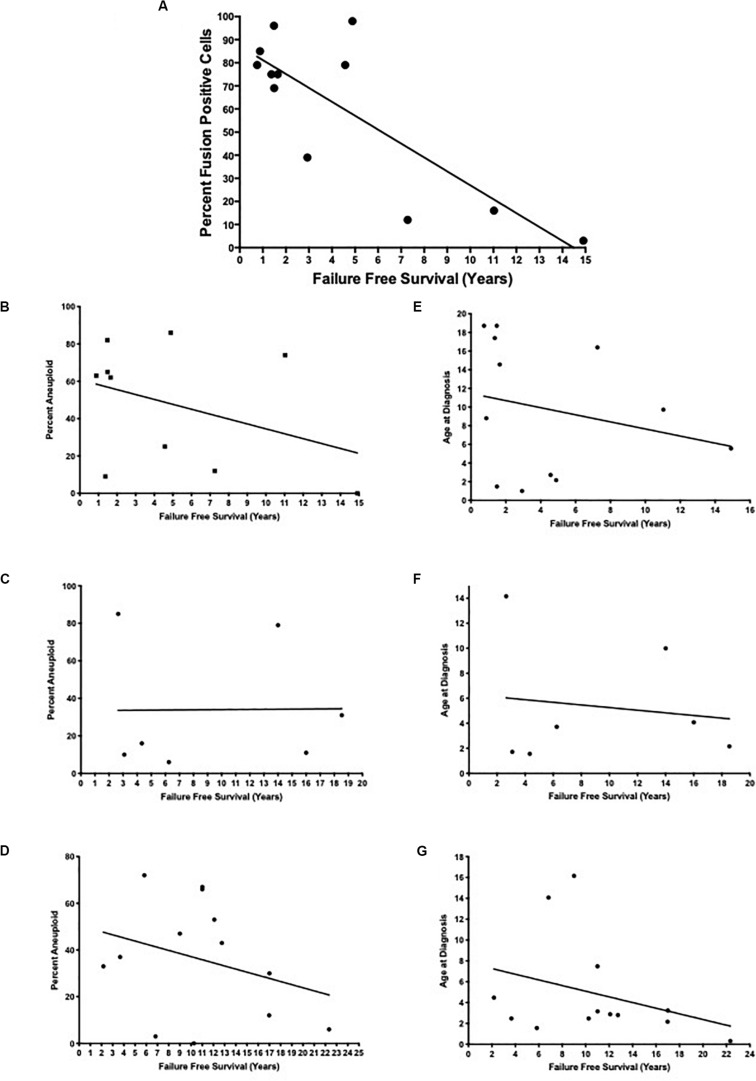
Examination of percent fusion positive cells, percent aneuploidy in cells, and age at diagnosis relative to failure-free survival. The percent intratumoral fusion positive cells **(A)** or the percent aneuploidy at PAX3, PAX7, and FOXO1 loci **(B–D)** were determined using our FISH probes, as described in the section “Materials and Methods.” The age at diagnosis **(E–G)** along with failure-free survival were obtained from a retrospective chart review and was calculated as the years between the initial diagnosis to death or to last scheduled appointment. The lines represent a linear regression of each set of data with the following statistics: **(A)** *R*^2^ = 0.66, *p* = 0.001; **(B)** *R*^2^ = 0.147, *p* = 0.273; **(C)** *R*^2^ = 0.00012, *p* = 0.982; **(D)** *R*^2^ = 0.091, *p* = 0.317; (E) *R*^2^ = 0.053, *p* = 0.450; (F) *R*^2^ = 0.021, *p* = 0.756; (G) *R*^2^ = 0.100, *p* = 0.292.

More importantly, when we examined the fusion positive ARMS patients more closely, we found a statistically significant inverse correlation between the extent of translocation positive intratumoral heterogeneity and failure-free survival rate (Table 2 and Figure 2A; *R*^2^ = 0.66, *p* = 0.001). Patients with a higher percentage of fusion positive containing cells presented with a higher stage and clinical grade of tumor, higher incidence of metastasis, a higher incidence of mortality and shorter failure-free survival rates relative to those with fewer fusion positive containing cells. We found no statistically significant correlation in clinical outcomes with respect to aneuploid-dependent intratumoral heterogeneity (fusion positive ARMS: *R*^2^ = 0.147, *p* = 0.273; fusion negative ARMS: *R*^2^ = 0.00012, *p* = 0.982; or ERMS: *R*^2^ = 0.091, *p* = 0.317) (Figures 2B–D), or age at initial diagnosis (fusion positive ARMS: *R*^2^ = 0.058, *p* = 0.450; fusion negative ARMS: *R*^2^ = 0.021, *p* = 0.756; ERMS: *R*^2^ = 0.100, *p* = 0.292) (Figures 2E–G).

## Discussion

Rhabdomyosarcoma (RMS) is one of the most common pediatric soft tissue sarcomas, comprised of two basic subgroups, alveolar (ARMS) and embryonal (ERMS). ARMS may be further subcategorized by whether it contains the (2;13) or the (1;13) chromosomal translocation (fusion positive) or whether they are fusion negative (Parham, 2001; Parham and Barr, 2013). At present tumors are diagnosed primarily by histologic or pathologic characterization with occasional use of RT-PCR or FOXO1 break-apart probes to determine the qualitative presence of a chromosomal translocation. However, these methods fall short in providing complete diagnostic information since FOXO1 dependent break apart probes are capable of determining the presence but not nature of the translocation while RT-PCR is capable of determining the nature but not extent of intratumoral translocation heterogeneity. The fact that various subclasses of RMS have different levels of aggressiveness (Galili et al., 1993; Davis et al., 1994; Epstein et al., 1995; Arnold et al., 2016) highlights the necessity of having a more accurate yet equally as simple diagnostic tool.

Along these lines, the Children’s Oncology Group published a report investigating the outcomes of patients with *t*(1;13) and *t*(2;13) positive ARMS. Their analysis determined that patients with *t*(2;13) containing tumors had a decreased survival and increased resistance to chemotherapy relative to patients with *t*(1;13) containing tumors. In addition, recent literature reports demonstrated that the fusion gene status and not other factors (e.g., histology, location of primary tumor) is the primary prognostic factor in the risk stratification of these patients (Burke et al., 2007; Missiaglia et al., 2012; Selfe et al., 2017). Taken together, these results strongly support the idea of placing a higher importance on determining the molecular status of RMS tumors (i.e., fusion gene status) in the risk stratification of these patients (Sorensen et al., 2002; Missiaglia et al., 2012). As an extension of this idea, in this work we utilized probes that allowed the simultaneous determination of the presence or absence of the *t*(1;13) and *t*(2;13) translocation in a cohort of RMS patient samples. We demonstrate that ARMS tumors contain intratumoral heterogeneity with respect to the presence or absence of the translocation event and that the extent of this heterogeneity inversely correlates to patient prognosis. These data provide yet another molecular diagnostic level that should be considered when determining prognostic stratification of RMS for the planning and management of these patients.

Other factors in addition to translocation status have been implicated in providing prognostic value for RMS patients, including the presence of aneuploidy, site of original tumor, and age at initial diagnosis. However, our retrospective analysis determined that there was no statistically significant correlation between survival and age at initial diagnosis (Figure 2) or site of original tumor (data not shown). In addition, aneuploidy is known to contribute to the increased aggressiveness of tumors, since chromosome instability and aneuploidy are potential mechanisms by which tumors generate their heterogeneity and adapt to selective growth pressure (Gordon et al., 2012). As with translocation status we observed intratumoral heterogeneity with respect to the presence or absence of aneuploidy of chromosomes 1, 2, and 13 (Table 1). However, our data did not demonstrate a statistically significant correlation between intratumoral heterogeneity for the presence or absence of aneuploidy and failure-free survival (Figure 2). We also saw a similar variation in aneuploid heterogeneity in fusion negative ARMS and ERMS tumor samples, patients who had much longer failure-free survival times and positive prognoses relative to patients with fusion positive ARMS (Table 1). Therefore, our results place a high significance on determining the molecular status of RMS tumors, consistent with previous reports (Burke et al., 2007; Missiaglia et al., 2012; Selfe et al., 2017) and extend these results to conclude that patient prognosis is also dependent on the extent of the presence or absence of a translocation event and not aneuploidy.

Our results and previous literature reports demonstrate that the presence or absence (Barr, 2009) along with the extent of heterogeneity of either *t*(1;13) or the *t*(2;13) (Figure 2) are consistent with a more aggressive tumor and poorer prognosis. Despite this knowledge, presently most biopsies rely primarily on histological classification of tumors with occasional qualitative determination of translocation status. Although histology is a reliable method for classifying RMS, it may sometimes misdiagnose these tumors, resulting in the use of less aggressive treatment methods and a poorer patient prognosis. This fact is evidenced in our results. We present two patients in our cohort, patients A and K, who were initially histologically diagnosed as being ERMS. Upon evaluation of their tumor samples using FISH probes capable of identifying both translocation events, it was found that these patients were, in fact, translocation positive with one containing 98% translocation positivity (Tables 1, 2), which alters their diagnosis and potential aggressiveness of treatment from ERMS to translocation positive ARMS. Therefore, we believe that it is essential to use molecular diagnostics that determine the presence and extent of both translocation events in conjunction with standard histological analysis to provide a more accurate diagnosis for patients presenting with RMS.

Finally, the ability to more accurately determine a prognosis based on translocation positive intratumoral heterogeneity will allow the use of more aggressive treatment methodology for those patients with the poorest outcomes. Consistent with this fact is the fortuitous treatment of Patient A in our cohort. This patient presented to Children’s Hospital in New Orleans with a stage 4, clinical grade 4, metastatic RMS that was originally histologically classified as ERMS. This patient was submitted to more aggressive treatment than normally used for ERMS, which included surgical resection of the primary tumor site followed by an extensive course of whole-body irradiation. Our FISH analysis determined that this tumor was not, in fact, ERMS but instead contained a 98% positive signal for the (2;13) translocation, classifying it solidly as ARMS with the worst prognostic outcome (Table 2). Due in part to the highly aggressive nature of the therapy, Patient A has so far had a failure-free survival rate over 5 years, unlike other ARMS patients in our cohort who had high percentages of translocation positivity and less aggressive therapies.

Taken together, our results along with the previous literature (Selfe et al., 2017) argue for utilizing the following strategy in the diagnosis and treatment of RMS patients. An initial histological analysis should be performed to classify the tumor as RMS. Following this classification, FISH analysis using probes that can simultaneously identify the presence of both translocation events will molecularly characterize the tumors as ARMS vs. ERMS. A subsequent quantitative analysis of the translocation positive cells will determine the extent of translocation heterogeneity, providing a risk stratification for these patients. Based on our results and the fortuitous treatment of Patient A, we then propose that patients with >70% translocation positivity would receive the most aggressive treatment, which could include surgical resection, standard chemotherapy, combined with full body irradiation. While seemingly excessive this aggressive treatment increased the survival of Patient A, who is still alive today.

## Conclusion

In conclusion, we demonstrate that intratumoral heterogeneity exists in ARMS with respect to the presence or absence of the (1;13) or (2;13) chromosomal translocation and that the extent of this heterogeneity has a statistically significant inverse correlation to patient outcome, as determined by failure-free survival. Taken together, these results provide a strong argument, building off previous reports, for the inclusion of these molecular analytic methods in biopsies, which when used in conjunction with standard histological analyses, may provide a more accurate diagnostic and prognostic tool for patients who present with RMS. This more accurate diagnosis then has the potential to indicate the use of more aggressive or specialized therapies to hopefully improve RMS patient survival.

## Data Availability Statement

The raw data supporting the conclusions of this article will be made available by the authors, without undue reservation, to any qualified researcher.

## Author Contributions

KG assisted in providing access to patient medical records, performed the initial retrospective chart review, and wrote the initial draft of the manuscript. JP performed the FISH analysis of tumor samples, analyzed the tumor slides, and assisted with the retrospective chart review. DM and AU assisted with performing and analyzing the tumor sample FISH results. JB assisted with the retrospective chart review. MS headed the tumor sample identification and preparation of the slides for the FISH analysis. FT co-directed the project, assisted in the analysis of the tumor sample FISH data, and assisted in the writing of the manuscript. AH co-directed the project, assisted in the overall data analysis, and wrote the final draft of the manuscript. All authors contributed to the article and approved the submitted version.

## Conflict of Interest

The authors declare that the research was conducted in the absence of any commercial or financial relationships that could be construed as a potential conflict of interest.
